# Detection of Rat Lungworm (*Angiostrongylus cantonensis*) in Rats and Gastropods, Italy

**DOI:** 10.3201/eid3109.250648

**Published:** 2025-09

**Authors:** Divakaran Pandian, Anna Šipková, Stefano Scarcelli, Giovanni Sgroi, Jana Kačmaříková, Francesco Buono, Elisa Castaldo, Nicola D’Alessio, Barbora Červená, Vincenzo Veneziano, David Modrý

**Affiliations:** Czech University of Life Sciences, Prague, Czech Republic (D. Pandian, D. Modrý); Masaryk University, Faculty of Science, Brno, Czech Republic (A. Šipková, D. Modrý); University of Naples Federico II, Naples, Italy (S. Scarcelli, F. Buono, E. Castaldo, V. Veneziano); Experimental Zooprophylactic Institute of Southern Italy, Portici, Italy (G. Sgroi, N. D’Alessio); University of Veterinary Sciences Brno, Brno (J. Kačmaříková, B. Červená); Institute of Vertebrate Biology, Czech Academy of Sciences, Brno (J. Kačmaříková, B. Červená); Biology Center of Czech Academy of Sciences, Institute of Parasitology, České Budějovice, Czech Republic (D. Modrý)

**Keywords:** Angiostrongylus cantonensis, rat lungworm, parasites, zoonoses, meningitis/encephalitis, neural angiostrongyliasis, emerging, Italy

## Abstract

The emerging zoonotic nematode *Angiostrongylus cantonensis* causes severe neural angiostrongyliasis in both humans and animals. The parasite has been reported in Spain. We detected *A. cantonensis* in rats and gastropods from the Campania region, southern Italy, demonstrating its broad distribution on the southern coast of Europe.

The rat lungworm, *Angiostrongylus cantonensis*, a neurotropic zoonotic parasite, is receiving increasing attention because of its potential to cause severe neurologic disease in humans and animals (*1*). This rat lungworm has an indirect life cycle involving rats (mainly *Rattus* spp.) as definitive hosts, mollusks as intermediate hosts, and different paratenic and transient hosts such as frogs, lizards, and crustaceans (*2*). Infection in humans usually occurs by accidental ingestion of infective third-stage larvae (L3) found in raw or undercooked snails or paratenic hosts or by contact with L3-contaminated water or products (*3*). Identified in China in 1935, *A. cantonensis* has since become endemic in Southeast Asia, East Asia, North and South America, and selected Pacific and Caribbean islands, where most human cases of neuroangiostrongyliasis occur; >7,000 human cases have been recorded worldwide (*4*).

In the past 2 decades, the geographic range of *A. cantonensis* lungworms has increased in Europe, and they have been detected in the Canary Islands (Tenerife, Spain) (*5*), in the Balearic Islands (Mallorca, Spain) (*6*), and most recently in mainland Spain (Valencia) (*7*), indicating a continued spread in the Mediterranean basin. Although human cases remain rare in Europe and have been associated with travel to well-established endemic regions, such as Southeast Asia and the Caribbean Islands (*8*), the subtropical climate and historically active maritime trade in Naples, Italy, provide favorable conditions for the spread of *A. cantonensis* to human and animal hosts. We investigated *Rattus* spp. rats and snail populations in periurban and rural areas of the Campania region of southern Italy to determine whether the *A. cantonensis* lungworm has spread to this region along the Mediterranean Coast of Europe.

## The Study

**Figure 1:**
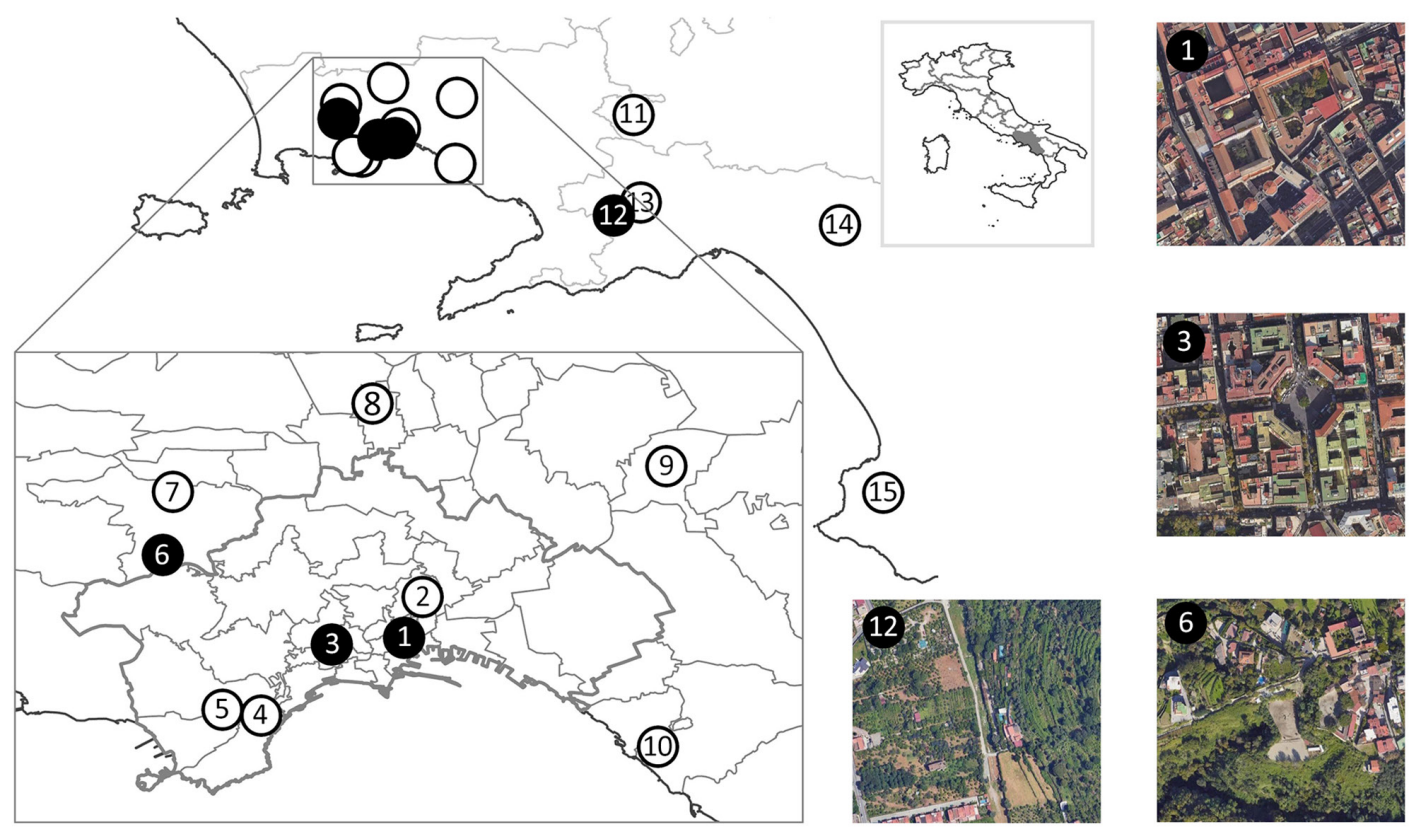
Sampling locations used for detection of rat lungworm (*Angiostrongylus cantonensis*) in rats and gastropods, Italy. Solid black circles indicate sites from which *A. cantonensis*–positive samples were collected, white circles indicate *A. cantonensis*–negative sites; numbering corresponds to numbers in the Table. Inset at top shows region of Italy in which sampling was conducted (gray area); inset at bottom shows detailed sampling areas within Naples; outer satellite images show areas with *A. cantonensis*–positive samples. Detailed information on locations, including geospatial positioning coordinates, are available in Appendix 1 Table. Map images created by using Google Maps (https://www.google.com/maps), Maxar Technologies (https://www.maxar.com), and Airbus (https://www.airbus.com).

We obtained a total of 32 frozen rat specimens, 10 *R. rattus* and 22 *R. norvegicus*, from a pest control company operating in metropolitan Naples and its surroundings. We conducted an initial sampling phase randomly across various locations. After we detected *A. cantonensis* lungworm in rats, we conducted a second sampling, during which we collected 352 gastropods from locations where infected rats were collected and from nearby areas selected at random. In total, we sampled rodent and gastropod samples from 15 locations during January–November 2024 (Figure 1; Appendix 1).

We necropsied rats at the Experimental Zooprophylactic Institute of Southern Italy in Naples. We isolated the heart and lungs and inspected them for adult *Angiostrongylus* spp. worms, characterized by the distinctive barber pole appearance in female nematodes; we preserved isolated worms in 96% ethanol. We froze tissue samples from the brain, heart, kidneys, liver, lungs, and spleen of rats for molecular analysis. We confirmed rat species on the basis of DNA extracted from spleen samples by using the Nucleic Acid Extraction Kit (Magnetic Bead Method) (Zybio, https://www.zybio.com), followed by amplification of the mitochondrial cytochrome b gene (Appendix 2). We detected *A. cantonensis* nematodes in 13 (40.6%) of 32 rats collected from 3 locations, with a mean of 7 (range 1–24) worms per rat (Table; Figure 1; Appendix 1). 

**Table:** Summary of rat and gastropod samples positive for rat lungworm (*Angiostrongylus cantonensis*), Italy

Location no.	Municipality (quarter)	Rats			Gastropods		GenBank accession no.
		No. positive/total no.	Haplotype		No. positive/total no.	Haplotype	
1	Naples (Porto)	1/1	NAP1		0/15	–	PV425925
2	Naples (San Carlo all'Arena)	0/1	–		0/61	–	–
3	Naples (Vomero)	1/3	NAP2		0/0	–	PV425926
4	Naples (Posillipo)	0/2	–		0/0	–	–
5	Naples (Fuorigrotta)	0/1	–		0/0	–	–
6	Naples (Camaldoli)	11/13	NAP1		7/73	NAP1	PV425925
7	Marano di Napoli	0/0	–		0/25	–	–
8	Casandrino	0/1	–		0/0	–	–
9	Casalnuovo di Napoli	0/0	–		0/12	–	–
10	Ercolano	0/1	–		0/0	–	–
11	Lauro	0/0	–		0/25	–	–
12	Corbara	0/4	–		1/76	†	–
13	Nocera Inferiore	0/0	–		0/51	–	–
14	Giffoni Valle Piana	0/0	–		0/14	–	–
15	Laureana Cilento	0/5	–		0/0	–	–

*Location numbers correspond to numbers in Figure 1. Detailed information on species and georeferenced data on sampled locations provided in Appendix 1 Table. ­–, no positive samples. †Failed sequencing.

**Figure 2:**
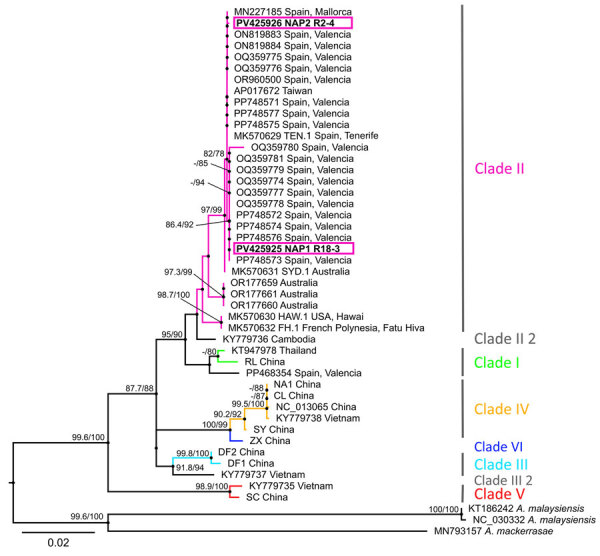
Maximum-likelihood phylogenetic tree of rat lungworm (*Angiostrongylus cantonensis*) detected in rats and gastropods, Italy. Tree is based on cytochrome c oxidase subunit 1 gene (1,578 bp) and partial sequences (215–561 bp) calculated by a Tamura-Nei plus model in IQ-TREE (http://www.iqtree.org) (*9*). Labeling of the clades follows previous studies (*10*). Sequences are labeled by GenBank accession numbers, where available, and locality of origin is indicated. Pink boxes indicate the 2 unique sequences from this study. Numbers at nodes indicate percentage SH-aLRT/ultrafast bootstrap support. Only values >75 are shown. Scale bar indicates nucleotide substitutions per site.

Molecular analysis confirmed *A. cantonensis* nematodes in 12 positive rats, and we subsequently sequenced 69 adult worms. We extracted DNA from those adult worms by using the DNeasy Blood & Tissue Kit (QIAGEN, https://www.qiagen.com), and we amplified the complete cytochrome c oxidase subunit 1 (*CO1*) gene (Appendix 2). The obtained sequences revealed 2 distinct haplotypes (NAP1 and NAP2), differing by 2 single-nucleotide polymorphisms (SNPs) at positions 1092 and 1481 of the *CO1* gene; the SNP at position 1481 resulted in a different amino acid. In a maximum-likelihood phylogenetic tree (Figure 2), both haplotypes clustered within clade II sensu, as previously defined (*9*), alongside other sequences from Europe, except for 1 (GenBank accession no. PP468354; 215 bp) from Valencia that clustered in a separate clade, a sister to clade I, differing from all other sequences from Europe in 3 SNPs. Compared with the TEN.1 isolate (GenBank accession no. MK570629) from Tenerife, Spain, each haplotype from Italy differed by a single SNP: NAP1, detected in Naples (Porto) and Marano di Napoli, differed at position 1092; and NAP2, detected in Naples (Vomero), differed at position 1481. The 394-bp sequence from Mallorca, Spain (GenBank accession no. MN227185), was identical to NAP2. Among the Valencia isolates, 10 sequences were identical to NAP1, 3 were identical to NAP2, and the rest differed in 1, 2, or 3 SNPs from the other sequences from Italy.

Collected gastropods were identified to species level by a trained malacologist on the basis of morphological criteria. Molecular identification was performed on juvenile and shell-less specimens lacking distinct morphological characteristics. DNA was extracted from muscle tissue using the same protocol used for rat spleen samples, with an extended overnight prelysis phase at 56°C, optimized for the L3 stage of *A. cantonensis*. Molecular identification was made on the basis of sequences of the mitochondrial 16S rRNA gene (Appendix 2). We detected *A. cantonensis* worms by using a species-specific quantitative PCR on DNA isolated from gastropod tissue (*11*). Of the 352 gastropods examined, 8 (2.3%) gastropods from 2 localities tested positive for *A. cantonensis* DNA (Figure 1, Table; Appendix 2). We successfully obtained 6 *CO1* gene fragment sequences from the 8 positive gastropods (Appendix 2), and compared those with sequences from adult *A. cantonensis* lungworms from rats in this study. All sequences belonged to the NAP1 haplotype.

## Conclusions

We provide robust evidence that the *A. cantonensis* rat lungworm is in the central Mediterranean region in the Naples area of Italy. Circulation of this zoonotic nematode in the highly populated Naples metropolitan area is concerning because of its ability to cause severe neurologic and ocular disorders in humans. Because Naples has an environment ideal for *A. cantonensis* transmission to the human population, enhanced awareness is needed among healthcare practitioners and diagnostic protocols should be revised and applied locally in the differential diagnosis of meningoencephalitis cases (*12*). In addition, considering reported clinical cases in domestic animals and in wildlife in known endemic foci (*13*–*15*), veterinary practitioners in the Naples area should be alerted.

Appendix 1 List of examined organisms with georeferenced localities and sequencing results from detection of rat lungworm (*Angiostrongylus cantonensis*) in rats and gastropods, Italy.

Appendix 2 Additional information on detection of rat lungworm (*Angiostrongylus cantonensis*) in rats and gastropods, Italy.
