# Supplementary material for: Detection of Rat Lungworm (Angiostrongylus cantonensis) in Rats and Gastropods, Italy
Source: Emerg Infect Dis. 2025 Sep;31(9):1833–7. doi: 10.3201/eid3109.250648 (PMC12407194; doi:10.3201/eid3109.250648)
Supplement: Appendix 2 — Additional information on detection of rat lungworm (Angiostrongylus cantonensis) in rats and gastropods, Italy. [file 25-0648-Techapp-s2.pdf]

Article DOI: https://doi.org/10.3201/eid3109.250648

EID cannot ensure accessibility for supplementary materials supplied by authors. Readers who have difficulty accessing supplementary content should contact the authors for assistance.

Detection of Rat Lungworm (*Angiostrongylus cantonensis*) in Rats and Gastropods, Italy

Appendix 2

PCR Assays

To amplify mitochondrial cytochrome b (CytB) and rRNA (16S) genes used in this study, the reaction consisted of 12.5 µL PCRBIO 2x Taq Mix Red (PCRBIO, London, UK), 1.25 µL each of 10 µM primers and 2 µL of extracted DNA in a total volume of 25 µL (Appendix 2 Table 1). The cycling conditions for amplifying complete cytochrome c oxidase subunit 1 (COI) gene from *A. cantonensis adults* obtained from rats and fragment of COI gene from *A. cantonensis* larvae from gastropods consisted of a reaction of 12.5 µL PCRBIO 2x Taq Mix Red (PCRBIO, London, UK), 1.25 µL each of 10 µM primers and 5 µL of extracted DNA in a total volume of 25 µL (Appendix 2 Table 2). PCR products were commercially sequenced in both directions using Sanger sequencing (Macrogen Europe, Amsterdam, Netherlands). All sequences were processed using Geneious Prime 2025.0.3 software (www.geneious.com).

Methodology of Applied Phylogenetics

Obtained sequences were checked for quality and trimmed manually using Geneious Prime v.2024.0.7 (https://www.geneious.com). The identity of sequences was validated using the BLAST software (Basic Local Alignment Search Tool (*6*)). For the phylogenetic analyses, available sequences of complete gene for subunit I of cytochrome c oxidase (COI) and any partial COI sequences originating from Europe were downloaded from the GenBank or from supplementary file to the relevant publication (*7*). Sequences of complete COI of *A. mackerrasae* and *A. malaysiensis* were used as the outgroup. These sequences were aligned with our

sequences representing unique haplotypes by Clustal Omega implemented in Geneious Prime. The maximum-likelihood phylogenetic tree was calculated by IQ-TREE (*8*). The most suitable model was chosen by ModelFinder (*9*) implemented in IQ-TREE based on the highest Bayesian information criterion scores and weights (BIC). The tree topology was tested by 1000 replicates of ultrafast bootstrap (*10*) and Shimodaira-Hasegawa (SH)-like approximate likelihood ratio test (*11*). The tree was then visualized in FigTree (http://tree.bio.ed.ac.uk/software/figtree) and edited in Inkscape (https://inkscape.org).

Appendix 2 Table 1. Cycling conditions for amplifying mitochondrial cytochrome b (CytB) and rRNA (16S) genes used in this study

Primer ID	Primer sequence (5‘-3‘)	Amplicon, bp	Cycling conditions	Reference
L14723<br>H15915	ACCAATGACATGAAAAATCATCGTT<br>TCTCCATTTCTGGTTTACAAGAC	1140	Initial denaturation 1‘ at 95°C; 40x 15“ at 95 °C denaturation, 15“ at 50 °C annealing, 1‘ at 72 °C extension with final extension 3‘0 at 72 °C.	(*1*)<br>(*1*)
16Sar<br>16Sbr	CGCCTGTTTATCAAAAACAT<br>CCGGTCTGAACTCAGATCACGT	400	Initial denaturation 1‘at 95°C; 35x 15“ at 95 °C denaturation, 15“ at 55 °C annealing, 8“ at 72 °C extension with final extension 3‘0 at 72 °C.	(*2*)<br>(*2*)

Appendix 2 Table 2. Cycling conditions for amplifying complete cytochrome c oxidase subunit 1 (COI) gene from *A. cantonensis* adults obtained from rats and fragment of COI gene from *A. cantonensis* larvae from gastropods in this study. Reaction consisted of 12,5 µL PCRBIO 2x Taq Mix Red (PCRBIO, London, UK), 1,25 µL each of 10 µM primers and 5 µL of extracted DNA in a total volume of 25 µL.

Primer ID	Primer sequence (5‘-3‘)	Amplicon, bp	Cycling conditions	Reference
COI_F1_Lv	GGTGATTATAATGTTTAATG	1700	Initial denaturation 1‘ at 95°C; 40x 15“ at 95 °C denaturation, 15“ at 55 °C annealing, 25“ at 72 °C extension with final extension 5‘0 at 72 °C.	(*3*)
2057 R	GGATATGAACCTCTCAGACT			This study
HCO 2198	TAAACTTCAGGGTGACCAAAAAATCA		Sequencing primers	(*4*)
COI_R1_Lv	CGTAGGAACCGCAATAAC		Sequencing primers	(*3*)
JB3	TTTTTTGGGCATCCTGAGGTTTAT		Sequencing primers	(*5*)
1002 F	CGGTTCCTACGGGAGTGAAG	674	Initial denaturation 1‘at 95°C; 40x 15“ at 95 °C denaturation, 15“ at 54 °C annealing, 15“ at 72 °C extension with final extension 5‘0 at 72 °C.	This study
2057 R	GGATATGAACCTCTCAGACT			This study

References

1. Ducroz JF, Volobouev V, Granjon L. An assessment of the systematics of arvicanthine rodents using mitochondrial DNA sequences: evolutionary and biogeographical implications. J Mamm Evol. 2001;8:173–206. https://doi.org/10.1023/A:1012277012303

2. Palumbi SR, Martin A, Romano S, McMillan WO, Stice L, Grabowski G. The Simple Fool's Guide to PCR. Mānoa (HI): University of Hawai‘i at Mānoa; 2002.

3. Lv S, Guo YH, Nguyen HM, Sinuon M, Sayasone S, Lo NC, et al. Invasive Pomacea snails as important intermediate hosts of *Angiostrongylus cantonensis* in Laos, Cambodia and Vietnam: Implications for outbreaks of eosinophilic meningitis. Acta Trop. 2018;183:32–5. PubMed https://doi.org/10.1016/j.actatropica.2018.03.021

4. Folmer O, Black M, Hoeh W, Lutz R, Vrijenhoek R. DNA primers for amplification of mitochondrial cytochrome c oxidase subunit I from diverse metazoan invertebrates. Mol Mar Biol Biotechnol. 1994;3:294–9. PubMed

5. Bowles J, Blair D, McManus DP. Genetic variants within the genus Echinococcus identified by mitochondrial DNA sequencing. Mol Biochem Parasitol. 1992;54:165–73. PubMed https://doi.org/10.1016/0166-6851(92)90109-W

6. Altschul SF, Gish W, Miller W, Myers EW, Lipman DJ. Basic local alignment search tool. J Mol Biol. 1990;215:403–10. PubMed https://doi.org/10.1016/S0022-2836(05)80360-2

7. Tian X, Chen S, Duan L, Qian Y, Li H, Lv S. The global spread pattern of rat lungworm based on mitochondrial genetics. Pathogens. 2023;12:788. PubMed https://doi.org/10.3390/pathogens12060788

8. Trifinopoulos J, Nguyen LT, von Haeseler A, Minh BQ. W-IQ-TREE: a fast online phylogenetic tool for maximum likelihood analysis. Nucleic Acids Res. 2016;44:W232–5. PubMed https://doi.org/10.1093/nar/gkw256

9. Kalyaanamoorthy S, Minh BQ, Wong TKF, von Haeseler A, Jermiin LS. ModelFinder: fast model selection for accurate phylogenetic estimates. Nat Methods. 2017;14:587–9. PubMed https://doi.org/10.1038/nmeth.4285

10. Minh BQ, Nguyen MAT, von Haeseler A. Ultrafast approximation for phylogenetic bootstrap. Mol Biol Evol. 2013;30:1188–95. PubMed https://doi.org/10.1093/molbev/mst024

11. Anisimova M, Gil M, Dufayard JF, Dessimoz C, Gascuel O. Survey of branch support methods demonstrates accuracy, power, and robustness of fast likelihood-based approximation schemes. Syst Biol. 2011;60:685–99. PubMed https://doi.org/10.1093/sysbio/syr041
